# Development of a Machine Learning-Based Cysticidal Assay and Identification of an Amebicidal and Cysticidal Marine Microbial Metabolite against *Acanthamoeba*

**DOI:** 10.1128/spectrum.00077-22

**Published:** 2022-04-25

**Authors:** Brian Shing, Mina Balen, William Fenical, Anjan Debnath

**Affiliations:** a Center for Discovery and Innovation in Parasitic Diseases, Skaggs School of Pharmacy and Pharmaceutical Sciences, University of California San Diego, La Jolla, California, USA; b Center for Marine Biotechnology and Biomedicine, Scripps Institution of Oceanography, University of California San Diego, La Jolla, California, USA; Huazhong University of Science and Technology

**Keywords:** *Acanthamoeba*, amoeba, assay development, cysticidal, cysts, drug discovery, free-living amoeba, machine learning, natural antimicrobial products, trophozoites

## Abstract

Traditional cysticidal assays for *Acanthamoeba* species revolve around treating cysts with compounds and manually observing the culture for evidence of excystation. This method is time-consuming, labor-intensive, and low throughput. We adapted and trained a YOLOv3 machine learning, object detection neural network to recognize Acanthamoeba castellanii trophozoites and cysts in microscopy images to develop an automated cysticidal assay. This trained neural network was used to count trophozoites in wells treated with compounds of interest to determine if a compound treatment was cysticidal. We validated this new assay with known cysticidal and noncysticidal compounds. In addition, we undertook a large-scale bioluminescence-based screen of 9,286 structurally unique marine microbial metabolite fractions against the trophozoites of A. castellanii and identified 29 trophocidal hits. These hits were then subjected to this machine learning-based automated cysticidal assay. One marine microbial metabolite fraction was identified as both trophocidal and cysticidal.

**IMPORTANCE** The free-living *Acanthamoeba* can exist as a trophozoite or cyst and both stages can cause painful blinding keratitis. Infection recurrence occurs in approximately 10% of cases due to the lack of efficient drugs that can kill both trophozoites and cysts. Therefore, the discovery of therapeutics that are effective against both stages is a critical unmet need to avert blindness. Current efforts to identify new anti-*Acanthamoeba* compounds rely primarily upon assays that target the trophozoite stage of the parasite. We adapted and trained a machine learning, object detection neural network to recognize *Acanthamoeba* trophozoites and cysts in microscopy images. Our machine learning-based cysticidal assay improved throughput, demonstrated high specificity, and had an exquisite ability to identify noncysticidal compounds. We combined this cysticidal assay with our bioluminescence-based trophocidal assay to screen about 9,000 structurally unique marine microbial metabolites against A. castellanii. Our screen identified a marine metabolite that was both trophocidal and cysticidal.

## INTRODUCTION

*Acanthamoeba* spp. are free-living amebae that can be opportunistic and non-opportunistic pathogens commonly associated with *Acanthamoeba* keratitis, a severe corneal infection (1). Current *Acanthamoeba* keratitis treatment relies upon a combination of antimicrobials, but despite aggressive treatment, 10% of patients suffer recurrent *Acanthamoeba* keratitis (2). This recurrence has been suggested to be due to the resilience of cysts to antimicrobials, which allows viable cysts to persist through treatment to excyst at a later date (3, 4).

Currently, there is a lack of interest in the pharmaceutical industry to identify compounds with activity against *Acanthamoeba*. As such, there is a lack of innovation in cysticidal compound discovery. The current standard for screening compounds for cysticidal activity is to treat *Acanthamoeba* cysts with a compound of interest and manually observe for evidence of excystation, such as proliferating trophozoites or distinctive trails left in agar medium by trophozoites (5, 6). These cysticidal screening techniques are labor-intensive and have low throughput, which limits their utility and efficiency.

Improving automation for cysticidal assays could significantly enhance current screening capabilities for *Acanthamoeba* and accelerate the rate of discovery of cysticidal compounds. Recent advances in machine learning and object detection algorithms present a unique asset to be leveraged for automating cysticidal assays. Convolution neural network object detection algorithms, such as R-CNN, SSD, and YOLOv3, allow computers to locate and classify objects within an image (7). Coupling automated high-content imaging systems with these machine learning, object detection algorithms could allow for novel automated cysticidal assays. Microscopy images could be analyzed through these object detection algorithms to determine the change in trophozoite number over a period of time and identify if a compound is cysticidal. To demonstrate the utility of our cysticidal assay, we performed screening of a marine microbial natural products library to identify marine metabolites that are both trophocidal and cysticidal.

## RESULTS

### Trophozoite counting algorithm machine learning training.

**FIG 1:**
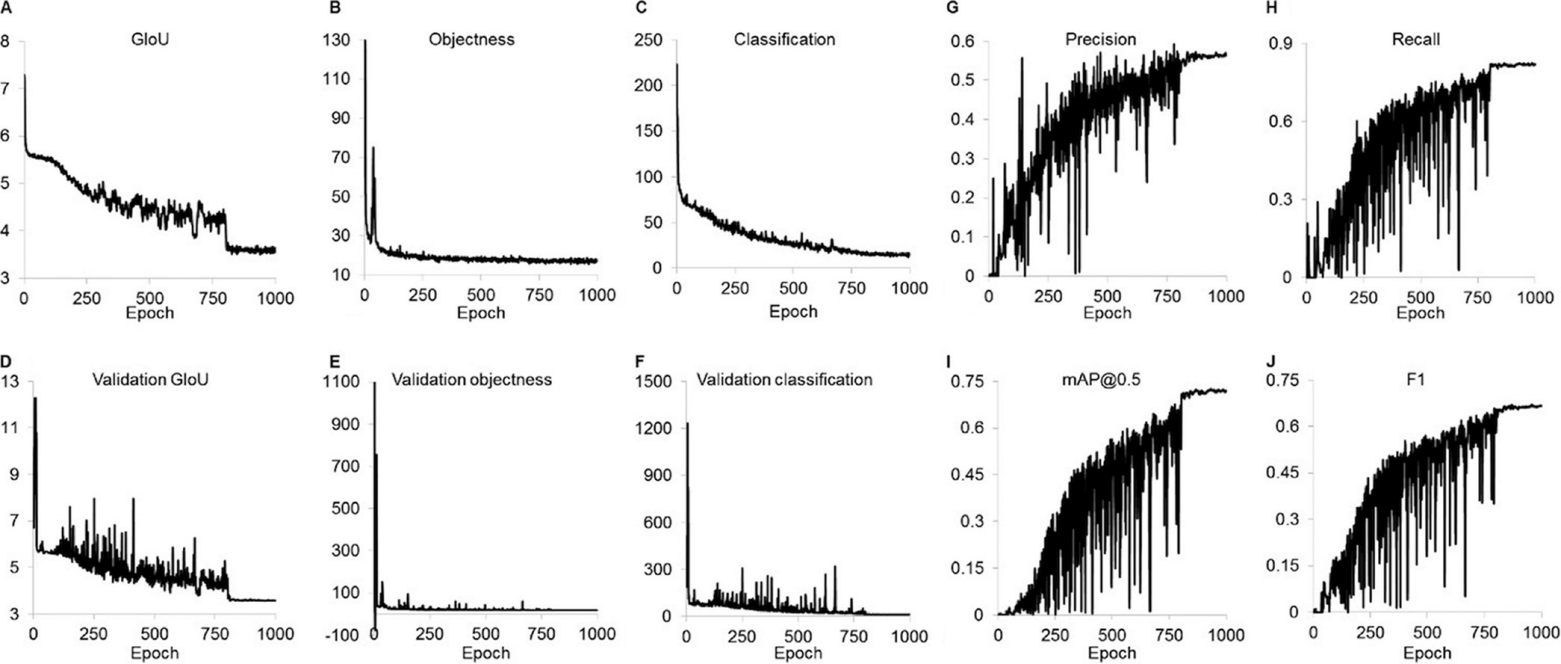
Training and validation metrics. Training and validation metrics were provided by YOLOv3 as a function of the training epoch. (A) Generalized intersection over union (GIoU) is a measure of the smallest box that encompasses both the predicted bounding boxes and actual ground truth boxes. (B) Objectness is the confidence a bounding box contains an object. (C) Classification is a measure of the accuracy of classifying objects in an image. (D) Validation GIoU is the GIoU for the validation set of images. (E) Validation objectness is the objectness score for the validation set. (F) Validation classification is the classification score for the validation set. (G) Precision = true positive/(true positive+false-positive). (H) Recall = true positive/(true positive+false-negative). (I) Mean average precision (mAP) for predictions with the intersection of union (IoU) >0.5 is the average precision for all classes (area under precision-recall curves for each class averaged). (J) F1 = 2×(precision x recall)/(precision + recall).

**FIG 2:**
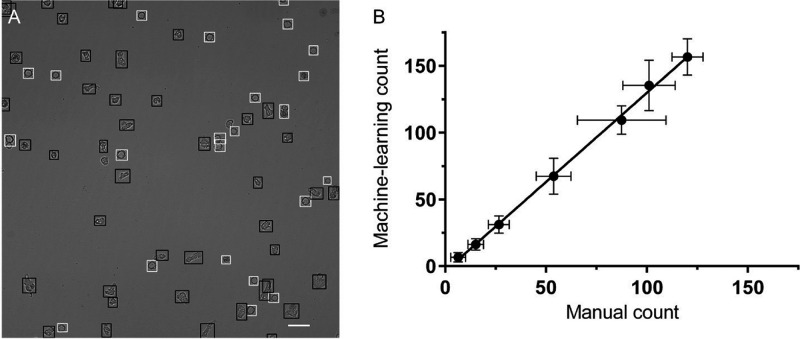
Evaluation of trophozoite and cyst cell count predictions. (A) Sample predictions of trophozoites and cysts 1 day after switching to PYG growth medium from encystation medium. Black boxes, detected trophozoites; white boxes, detected cysts. Magnification: 200×; Scale bar: 50 μm. (B) Linear correlation between manual (*x*-axis) and machine learning (*y*-axis) counts of seeded trophozoites; R^2^: 0.9984; *n* = 4.

A Google colaboratory environment was created to train a YOLOv3 object detection algorithm. The algorithm was trained for 1000 epochs on manually labeled images of cysts and trophozoites. Throughout the training, the algorithm gradually improved the identification of cysts and trophozoites within microscopy images. The precision, recall, F1, and mAP@0.5 increased monotonically and plateaued around 800 training epochs (Fig. 1G to J), indicating additional training past epoch 800 would not significantly improve the model’s performance. The final model (1000 epochs) used for inference mode to count the cysts and trophozoites had a precision of 0.563, recall of 0.821, F1 score of 0.665, and mAP@0.5 of 0.721 (Fig. 1G to J). Sample bounding boxes labeling trophozoites and cysts qualitatively demonstrate the object detection algorithm was able to identify cysts and trophozoites (Fig. 2A).

The counts of trophozoites as determined by the YOLOv3 algorithm were compared to manual counting techniques to determine if these techniques provided comparable cell counts. A. castellanii trophozoites were seeded at serially diluted concentrations ranging from 4.0 × 10^4^ to 625 trophozoites/100 μL. These trophozoites were imaged 1 h after seeding to minimize the effect of proliferation on cell numbers and accurately reflect the starting number of cells. Equivalence between the YOLOv3 algorithm and manual counting techniques was confirmed by linear correlation between trophozoites manually counted by light microscopy and YOLOv3 algorithm-derived counts (Fig. 2B).

### Cysticidal assay validation.

A cysticidal compound would result in non-viable cysts, which should not result in an increase in trophozoites even with incubation in the growth medium. A noncysticidal compound should have excystation and trophozoite proliferation after incubation in the growth medium. The trained YOLOv3 algorithm was utilized to count the number of trophozoites on days 0 and 3 following the removal of compounds and exchange with the PYG growth medium. These counts were analyzed to determine if a well of the plate displayed evidence of excystation and trophozoite proliferation.

This novel phenotypic screen was first validated with reference compounds. A. castellanii cysts were treated with literature-relevant dilutions of reference compounds chlorhexidine, alexidine, caspofungin, amphotericin B, corifungin, and fluconazole and imaged. The change in the number of trophozoites from day 0 to day 3 was determined for each well, and logistic regressions for each plate were calibrated using wells treated with 461.85 μM polyhexamethylene biguanide (PHMB) as positive-controls (cysticidal hits) and 0.5% (vol/vol) DMSO as negative-controls (noncysticidal). Wells with a probability of being cysticidal > 0.9 as determined by the logistic regression were considered to be cysticidal. Each reference compound was serially diluted 2-fold at concentrations relevant to previously reported literature values to assess their minimum cysticidal concentrations (Table 1).

**TABLE 1:** Comparison of YOLOv3 and manual determination of minimum cysticidal concentrations

Compound	YOLOv3 MCC^*a*^ (μM)	Manual MCC (μM)
Chlorhexidine	6.94 – 3.47	13.89 – 6.94
Alexidine	<10.74	<10.74
Corifungin	>1,600	>1,600
Amphotericin B	>138.52	>138.52
Caspofungin	>105.49	>105.49
Fluconazole	>6,686.91	>6,686.91

a MCC, minimum cysticidal concentration.

Alexidine has been classically reported to be cysticidal at 100 μg/mL (equivalent to 171.91 μM) (8). Manual observation determined all dilutions of alexidine were cysticidal, which suggests a minimum cysticidal concentration of <10.74 μM. The YOLOv3 algorithm successfully predicted the same minimum cysticidal concentration as the manual observation. While the minimum cysticidal concentrations as determined by manual observation and YOLOv3 are lower than literature reported values, this could be due to these results being taken on day 3 as opposed to a later time point.

Chlorhexidine has been previously reported to have a mean minimum cysticidal concentration of 7.02 μg/mL (equivalent to 13.89 μM) with a minimum cysticidal concentration ranging from 13.52 to 3.12 μg/mL (equivalent to 26.75 to 6.17 μM) (9). In this validation, chlorhexidine was manually verified to have a minimum cysticidal concentration between 13.89 and 6.94 μM. In contrast, the YOLOv3 predictions suggested a minimum cysticidal concentration between 6.94 and 3.47 μM.

Amphotericin B, caspofungin, fluconazole, and corifungin were not detected as cysticidal at any of the tested concentrations by both the YOLOv3 and manual observation. These findings are consistent with previous reports of amphotericin B, caspofungin, and fluconazole being noncysticidal (5).

Interestingly, corifungin was not cysticidal at any of the concentrations tested. The YOLOv3 predictions were consistent with manual observations that none of the tested concentrations proved to be cysticidal. These findings seem to contrast with previously reported observations of subcellular damage in A. castellanii cysts treated with high concentrations of corifungin (10). It was not clear whether this was due to the degradation of the stock compound or due to the use of a different strain.

While there were some variations in the minimum cysticidal concentration predicted by the YOLOv3 algorithm and manual observation for chlorhexidine, a closer analysis of the YOLOv3 predictions against the manual observations revealed this assay had high accuracy and specificity in predicting cysticidal and noncysticidal compounds at day 3.

The predictions from the YOLOv3 algorithm were compared against manual observations for excystation in each well on day 3 (Table 2). The YOLOv3 algorithm had an overall accuracy ([true positive + true negative]/[true positive + false-positive + true negative + false negative]) of 0.97. The algorithm also displayed a recall (true positive/[true positive + false negative]) of 1, specificity (true negative/[true negative + false positive]) of 0.96, and precision (true positive/[true positive + false positive]) of 0.90. The high specificity of this assay suggests it can accurately identify noncysticidal compounds.

**TABLE 2:** Confusion matrix on evaluation of validation screen

	Predicted Cysticidal	Predicted Noncysticidal	Total
Observed Cysticidal	109	0	109
Observed Noncysticidal	12	311	323
Total	121	311	432

### Screening of marine natural products library.

**FIG 3:**
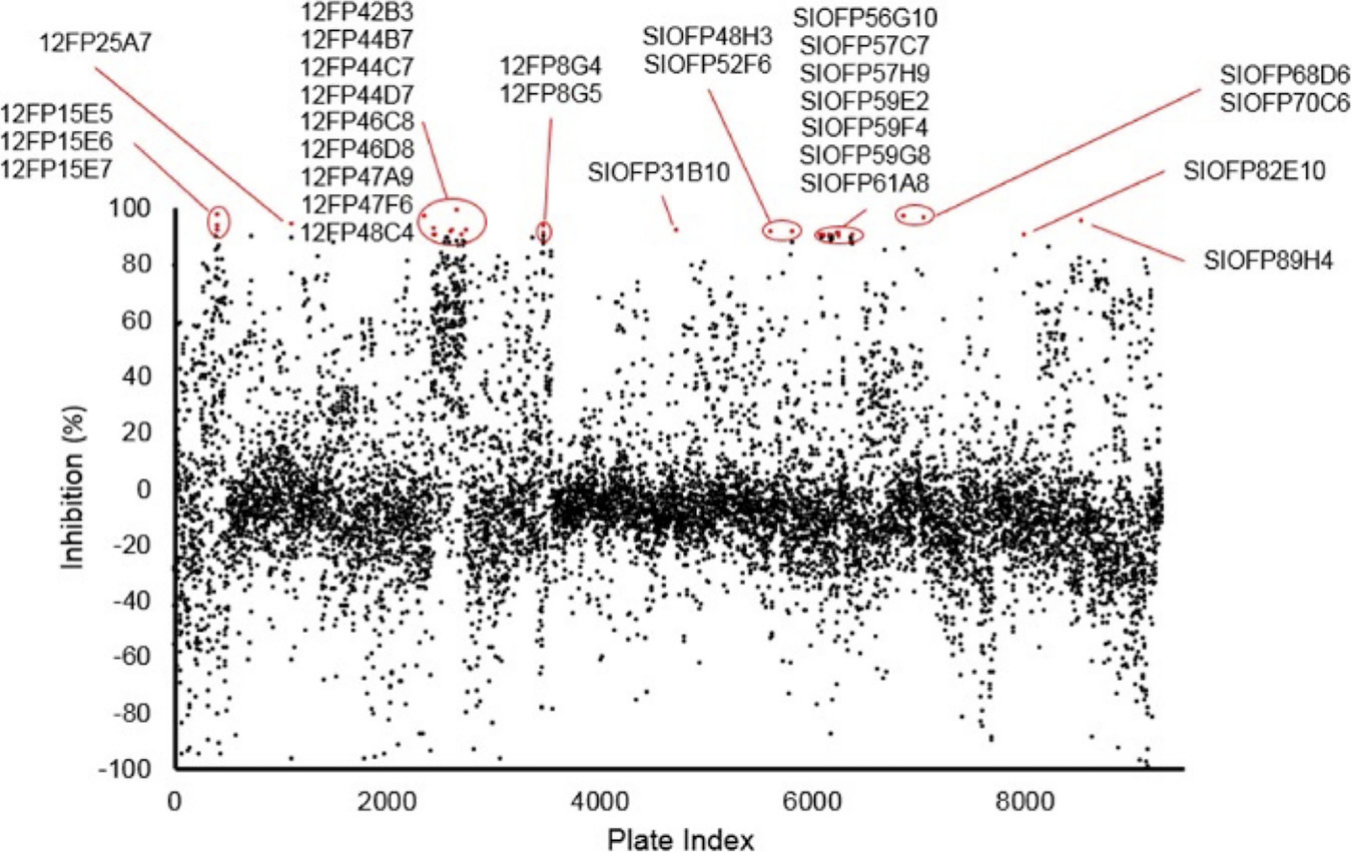
Normalized growth inhibition of marine fractions. Unique marine microbial natural products test fractions (9,286) were screened against A. castellanii trophozoites at 60 μg/mL. Black, fractions displaying <90% inhibition against A. castellanii trophozoites. Red, fractions displaying >90% inhibition against trophozoites.

**(i) Amebicidal assay.** A library of 9,286 unique marine natural products fractions was screened for activity against A. castellanii trophozoites at 60 μg/mL. 29 fractions displayed >90% inhibition and were selected for further secondary screening on cysts (Fig. 3).

**FIG 4:**
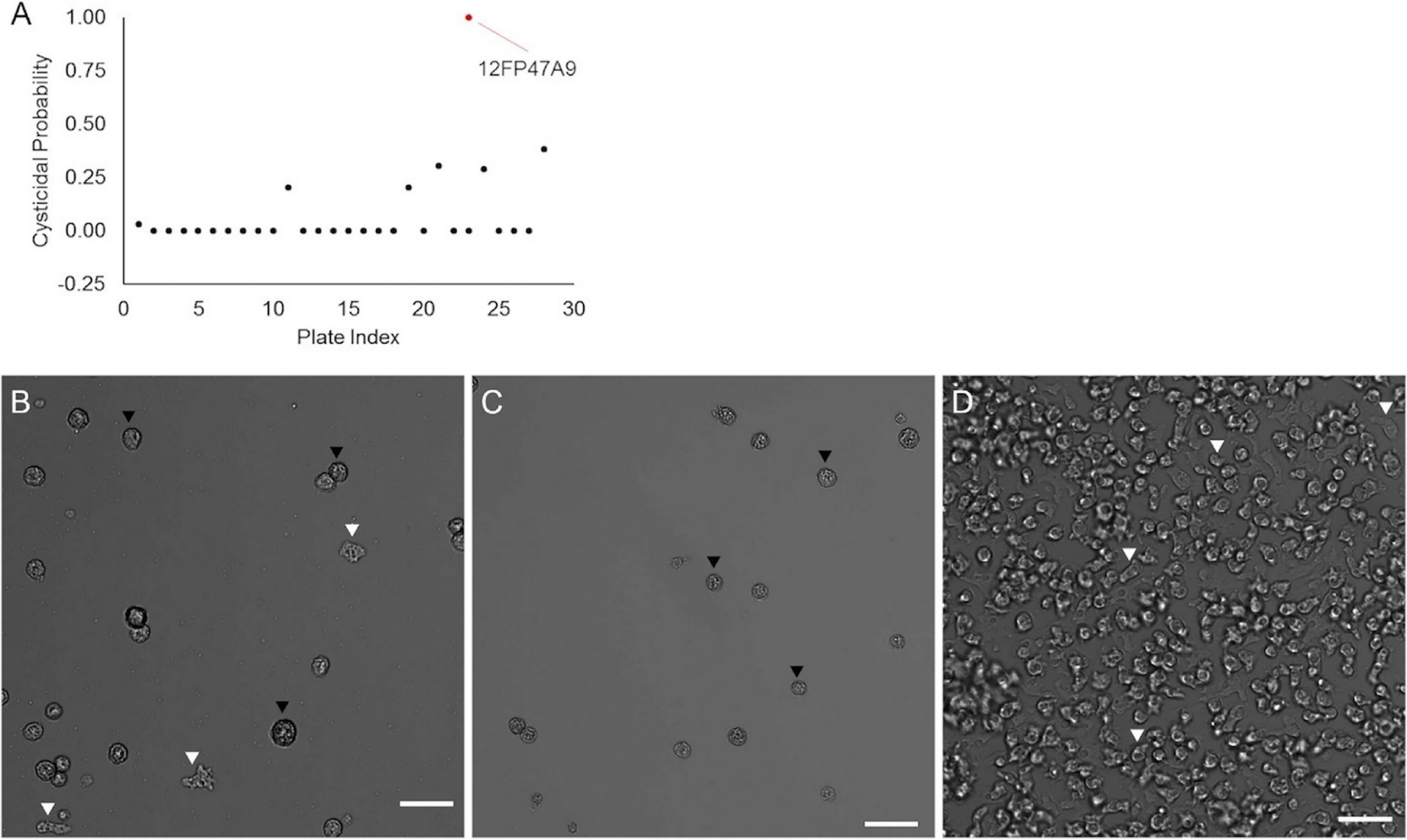
Cysticidal Screen. 29 fractions identified as having trophocidal activity were screened against A. castellanii cysts. (A) The probability of a treatment being cysticidal (*y*-axis) is plotted as a function of the fraction’s location on a plate (*x*-axis). Red, trophocidal hit 12FP47A9 flagged as cysticidal fraction; black, trophocidal hits that were not cysticidal. Microscopy images were taken on day 3 of incubation in the PYG growth medium. Wells were treated with (B) 120 μg/mL fraction 12FP47A9; (C) 923.71 μM (0.04% [wt/vol]) PHMB (positive-control); (D) 1% (vol/vol) DMSO (negative-control). Black arrowhead, cyst; white arrowhead, trophozoite; 200× magnification; scale bar: 50 μm.

**(ii) Cysticidal assay.** The 29 fractions identified in the primary screen were then evaluated against cysts at 120 μg/mL. Images from the fractions were processed through the YOLOv3 machine learning algorithm to determine the counts of trophozoites on days 0 and 3. After analyzing the change in trophozoites, fraction 12FP47A9 was identified as potentially cysticidial (Fig. 4A). This potentially cysticidal well was manually evaluated. On day 3, fraction 12FP47A9 displayed a minimal amount of excystation (Fig. 4B) while all other fractions and the negative-control (Fig. 4D) were confluent by day 3.

**(iii) Half-maximal effective concentration (EC_50_) determination.** The top 10 fractions displaying the highest inhibition against trophozoites were assessed to determine their EC_50_ values. The fractions had EC_50_ values ranging from 0.4 to >60 μg/mL (Table 3). Interestingly, these EC_50_ values had little to no correlation with the probability of the fraction being cysticidal, suggesting amebicidal activity was not a good predictor of cysticidal activity. Fraction 12FP47A9, which was the strongest candidate for being cysticidal, did not appear to have the strongest amebicidal activity of these fractions. Fractions with significantly lower EC_50_ values against trophozoites, such as 12FP15E5, were completely noncysticidal.

**TABLE 3:** Sources, EC_50_, and CC_50_ of Top 10 trophocidal fractions^*a*^

Fraction	Source/strain no.	Location/date	EC_50_ (μg/mL)			CC_50_ (μg/mL)		P^*c*^
			Mean	95% lower CL^*b*^	95% upper CL	HEK-293	HT-29	
12FP15E5	TAA522	Coarse sand, Channel Islands, 2012	0.4	0.1	2.2	ND^*d*^	ND	0
12FP15E6	TAA522	Coarse sand, Channel Islands, 2012	1.9	0.8	4.4	25	ND	0
12FP15E4	TAA522	Coarse sand, Channel Islands, 2012	3.0	1.3	6.6	ND	ND	0
12FP15E7	TAA522	Coarse sand, Channel Islands, 2012	4.9	2.5	9.4	ND	50	0
SIOFP89H4	CNT714	Sand, San Diego Bay, 2008	6.4	6.0	6.7	ND	ND	0
12FP25A7	TAA584	Coarse sand, Channel Islands, 2012	6.8	3.6	12.8	50	50	0
12FP47A9	TAA844	Sand, Marianas Islands, 2012	20.3	19.7	21.0	50	50	1.0
12FP42B3	TAA734	Sand, Marianas Islands, 2012	21.2	15.3	29.3	50	>50	0
SIOFP68D6	CNT284	Sponge, Bahamas, 2007	55.9	32.4	96.2	>100	>100	0
SIOFP70C6	CNT292	Sponge, Bahamas, 2007	>60			>100	ND	0

a Sources and EC_50_ concentrations of the top 10 fractions display the most inhibition against trophozoites. Few fractions were also selected for cytotoxicity testing on mammalian epithelial cells HEK-293 and HT-29.

b CL, confidence limit.

c P, the probability a fraction was cysticidal as determined by cysticidal assay.

d ND, not determined.

**(iv) Mammalian cytotoxicity assay.** Although compounds were not isolated from the fractions, we undertook a mammalian cytotoxicity study with few trophocidal fractions that exhibited low to high EC_50_ against A. castellanii. These fractions inhibited the growth of representative epithelial cells HEK-293 and HT-29 at half-maximal cytotoxic concentration (CC_50_) values of 25, 50, or >100 μg/mL (Table 3). Thus, these trophocidal fractions were about 2-, 7-, or 10-fold less toxic to human epithelial cells than A. castellanii.

### Determination of minimum cysticidal concentration of 12FP47A9.

**FIG 5:**
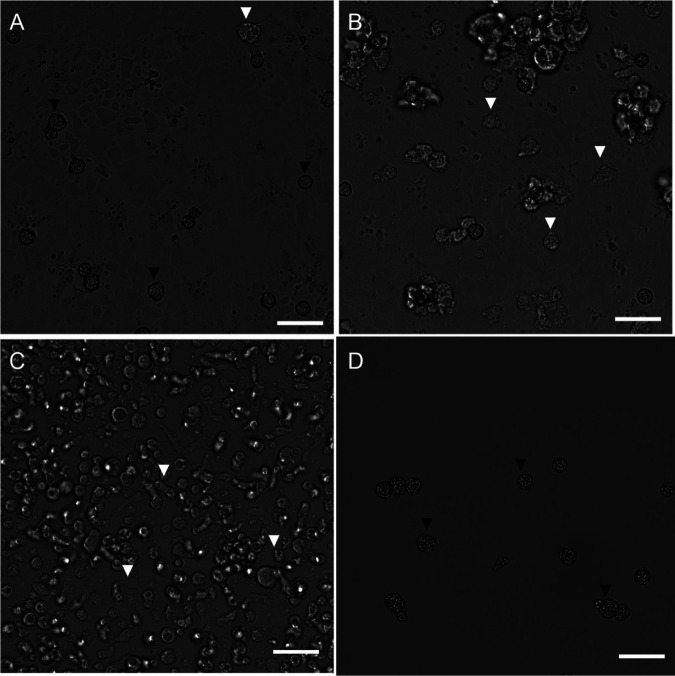
12FP47A9 Minimum cysticidal concentration determination. Microscopy taken on day 7 of incubation in PYG growth medium. Wells are treated with (A) 480 μg/mL 12FP47A9; (B) 240 μg/mL 12FP47A9; (C) 4% (vol/vol) DMSO (negative-control); (D) 0.16% (wt/vol) PHMB (positive-control). Black arrowhead, cyst; white arrowhead, trophozoite; 200× magnification; scale bar: 50 μm.

Because the fraction of 12FP47A9 demonstrated cysticidal activity, it was evaluated manually to determine its minimum cysticidal concentration. Cysts were treated with final concentrations of 12FP47A9 ranging from 480 to 3.75 μg/mL and evaluated for excystation and trophozoite growth for up to 7 days. Fraction 12FP47A9 demonstrated minimal to no excystation at 480 μg/mL (Fig. 5A). A lower concentration of 12FP47A9 (240 μg/mL) resulted in moderate excystation and trophozoite proliferation (Fig. 5B), suggesting a minimum cysticidal concentration between 480 and 240 μg/mL. These findings suggest fraction 12FP47A9 may provide promising anti-*Acanthamoeba* drug-like molecule candidates for further study.

## DISCUSSION

Current screening techniques for identifying cysticidal compounds against *Acanthamoeba* largely rely upon manual observation. Unfortunately, the manual nature of these assays can be a bottleneck that hampers the rate of cysticidal drug discovery. Machine learning and object detection algorithms are relatively new, albeit promising, technologies because these algorithms could be adapted to recognize specific cell types and take over the role of manual observation to significantly improve the rate of cysticidal drug discovery.

This novel cysticidal assay screens for cysticidal compounds under axenic cell culture conditions. For screening, potential cysticidal compounds were defined as having minimal to no excystation by day 3. Day 3 was considered to be the endpoint because cysts in wells treated with DMSO (negative-control) excysted out and became confluent with trophozoites by day 3. These wells became over confluent and individual trophozoites were difficult to distinguish past day 3.

This assay was validated with known cysticidal and noncysticidal compounds. These compounds were diluted serially and were screened by this assay. Predictions of the minimum cysticidal concentration of each compound by machine learning algorithm were compared to manual observations. Notably, the assay’s prediction of minimum cysticidal concentration of chlorhexidine was lower than what was manually observed by a factor of 2, suggesting the rare possibility for false-positives (i.e., predicting a treatment to be cysticidal when it is not). However, this assay displayed very high specificity and correctly identified all noncysticidal wells, suggesting it can accurately screen out noncysticidal compounds. This still provides major utility because this assay is amenable to high-throughput screening and could be used to screen out noncysticidal compounds in large compound libraries, leaving only a small amount of potentially cysticidal compounds for further manual evaluation.

Although natural products have been used for the treatment of parasitic diseases, none of them came from marine sources (11, 12). Drug discovery research for *Acanthamoeba* keratitis with marine natural products was only limited to a marine alga *Laurencia* sp. (13, 14). To identify novel marine metabolites with anti-*Acanthamoeba* activity, a marine natural products fraction library was evaluated to demonstrate the utility of this novel cysticidal assay. Because compounds useful for *Acanthamoeba* keratitis treatment should have dual activity against the trophozoite and cyst stages, these fractions were first screened against trophozoites of *Acanthamoeba*. Any compound that is not amebicidal would not be worth further consideration for investigation as trophozoites play an active role in *Acanthamoeba* keratitis.

**FIG 6:**
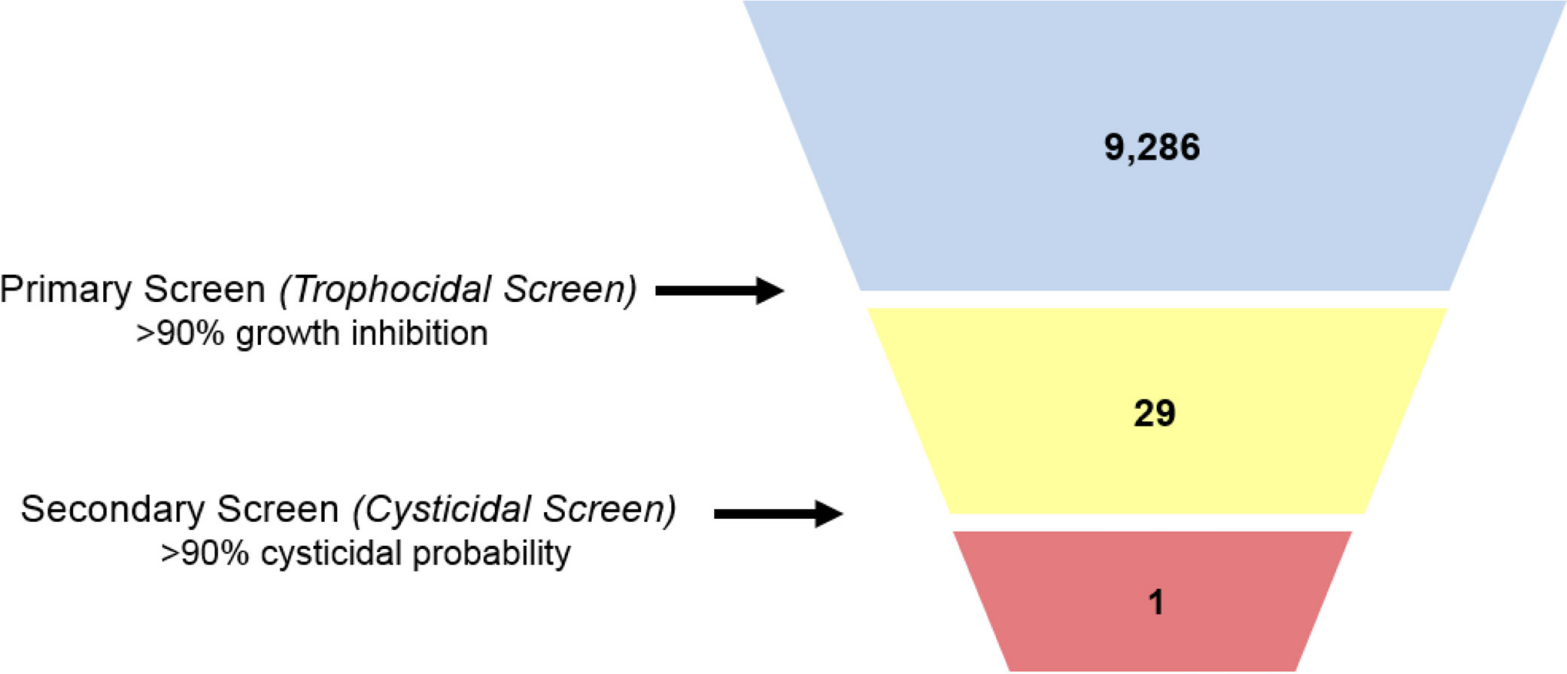
Screening summary. Overview of marine natural product library screen. Unique test fractions (9,286) were screened against trophozoites. Twenty-nine fractions displaying >90% inhibition against trophozoites were screened against cysts, and fraction(s) with >90% probability of being cysticidal were considered potentially cysticidal compounds.

The primary screen identified 29 fractions with high inhibition (>90%) against trophozoites. These fractions were then screened through this novel cysticidal assay, which identified fraction 12FP47A9 as potentially cysticidal (Fig. 6). At 120 μg/mL, fraction 12FP47A9 displayed minimal to no excystation by day 3, suggesting its potential as a cysticidal compound. This was followed with further manual evaluation, and 480 μg/mL displayed minimal to no excystation by day 7.

This new cysticidal assay helped identify a new potentially cysticidal fraction, which was subsequently verified by manual observation, demonstrating its utility as a cysticidal screen. Furthermore, this assay is useful because it can weed out noncysticidal treatments and is amenable to high-throughput screening. The lack of correlation between EC_50_ (amebicidal) and cysticidal data further demonstrates the need for cysticidal assays as amebicidal compounds are not necessarily cysticidal. While this assay utilized YOLOv3 for classifying, identifying, and counting trophozoites and cysts, machine learning and object detection algorithms are highly flexible and adaptable platforms that could be used to automate cell counting and for identification of other phenotypically distinct cells to improve any morphological cell studies.

Taken together, our machine learning-based cysticidal assay and screening of marine microbial metabolites identified an amebicidal and cysticidal fraction 12FP47A9. This fraction may harbor anti-*Acanthamoeba* molecules that would serve as attractive drug development lead molecules. Future studies will involve isolation of pure compounds, testing of compounds to identify the actives, and comprehensive assignment of structure.

## MATERIALS AND METHODS

### A. castellanii cell culture.

A. castellanii strain Ma trophozoites from the American Type Culture Collection (ATCC) (number 50370) were cultured and maintained at 28°C and 5% CO_2_ in peptone yeast glucose (PYG) medium supplemented with 100 U/mL penicillin and 100 μg/mL streptomycin.

### Culture of mammalian epithelial cells.

HEK-293 and HT-29 epithelial cells were maintained in DMEM medium, supplemented with penicillin (100 U/mL), streptomycin (100 μg/mL), and 10% heat-inactivated fetal bovine serum, and cultured according to ATCC specifications. The logarithmic phase of growth of the human cell lines was determined by counting the cells and all experiments were performed using human cells harvested during the logarithmic phase of growth.

### Cyst microplate preparation.

Cysts were prepared by culturing trophozoites in an encystation medium (95 mM NaCl, 5 mM KCl, 8 mM MgSO_4_, 0.4 mM CaCl_2_, 1 mM NaHCO_3_, 20 mM Tris-HCl, pH 9.0) (15). Trophozoites were collected by centrifugation at 200 g for 5 min and washed in PBS three times before resuspension in an encystation medium. Trophozoites (5 × 10^3^ in 99.5 μL of encystation medium) were seeded into each well of a 96-well microplate for 48 h to promote encystation. After 48 h, the cysts were treated with 0.5 μL of compounds of interest dissolved in DMSO in triplicate wells. 0.5% (vol/vol) DMSO served as negative-controls while 468.15 μM PHMB served as positive-controls. The cysts were incubated for 48 h with compounds of interest. Afterward, the encystation medium was removed, and the wells were washed four times with PBS before the addition of 100 μL of PYG medium. A. castellanii cysts were incubated in a PYG medium to promote excystation.

### Trophozoite counting algorithm machine learning training.

A Google colaboratory notebook environment was created for the ultralytics YOLOv3 object detection algorithm implementation downloaded from https://github.com/ultralytics/yolov3.

A. castellanii trophozoites were encysted as previously described and allowed to excyst for 24 h in a PYG growth medium. These cells were imaged, and the tiff microscopy images were converted in batches to jpg file format using a custom python script. The trophozoites and cysts in these images were hand-labeled and annotated using labelImg. The training set consisted of 58 images with 3598 trophozoites and 3937 cysts labeled. The test set consisted of 20 images with 2127 trophozoites and 2099 cysts labeled. The validation set consisted of 10 images with 814 trophozoites and 785 cysts labeled. These data sets were utilized to train a custom model. Setup was done per ultralytics YOLOv3 instructions for training on custom data within a Google colaboratory environment (16). The training was conducted in a Python 3 runtime environment with GPU acceleration and utilizing the provided yolov3-tiny.cfg configuration file. The model was trained for 1,000 epochs, and the last epoch model was utilized for object detection.

To determine equivalence between manual and the machine learning derived counts, trophozoites were serially diluted 2-fold from 4.0 × 10^4^ to 625 trophozoites and seeded into microplate wells in 100 μL of PYG growth medium and allowed to attach for an hour. The wells were then imaged by an ImageXpress Micro XLS (Molecular Devices). The images were inputted into the trained YOLOv3 model to count the number of trophozoites in each image. These counts were compared to the number of trophozoites in each image as determined by manual counting to assess equivalency.

### Cysticidal assay validation.

A microplate of cysts was generated following the microplate preparation method previously described. A. castellanii cysts were treated with serial 2-fold dilutions of compounds reported to be cysticidal or noncysticidal at concentrations relevant to previously reported literature.

Chlorhexidine gluconate (Acros Organics) was tested from 111.11 to 0.86 μM. Alexidine dihydrochloride (Sigma-Aldrich) was tested from 1,375.26 to 10.74 μM. Corifungin (Acea Biotech) was tested from 1,600 to 12.5 μM. Amphotericin B (Gold Bio) was tested from 138.52 to 1.08 μM. Caspofungin diacetate (Sigma-Aldrich) was tested from 105.49 to 0.82 μM. Fluconazole (Fluka Analytical) was tested from to 6,686.91 to 52.24 μM.

Each well of the microplate was imaged on the ImageXpress Micro XLS on day 0 and day 3 following the removal of the encystation medium with compounds and the addition of the PYG medium. The images were run through the YOLOv3 algorithm to count the number of trophozoites and determine the change in trophozoite number from day 0 to day 3. The Realstatistics Excel plugin was used to run logistic regressions with the change in trophozoites for 0.5% (vol/vol) DMSO serving as negative-controls while the change in trophozoite count for 461.85 μM PHMB served as positive-controls. Wells with a probability of being cysticidal >0.9 were defined as hits. Images were also viewed manually for evidence of excystation on day 3 and compared to the YOLOv3 predictions to generate a confusion matrix for assessing the accuracy, precision, recall, and specificity of the assay.

### Screening of marine natural products library.

**(i) Marine natural products library composition.**

The test samples in this study were derived from the collection, cultivation, and extraction of marine bacterial cultures. These bacteria, which are mainly members of the Actinomycetales, were collected from numerous locations worldwide (Table 3). The samples are test fractions derived from the chromatographic purification of 5 L culture whole ethyl acetate extracts. The test samples vary containing 5 to 8 polar to nonpolar materials in each.

**(ii) Amebicidal assay.** A marine natural products library of 9,286 unique fractions was assayed for anti-*Acanthamoeba* activity on trophozoites. A BioSero ATS (EDC Biosystems) was used to transfer 0.25 μL of each marine natural product fraction (12 mg/mL stock) or control into each well of Greiner Bio-One Cellstar white, flat bottom microplates. 0.5% (vol/vol) DMSO and 461.85 μM PHMB served as negative-controls and positive-controls, respectively. The fractions were tested in duplicate. Afterward, a Multidrop Combi liquid handler (Thermo Fisher) was used to transfer 50 μL of cell solution containing 5 × 10^3^ trophozoites into each well.

The plates were incubated at 28°C and 5% CO_2_ for 48 h before luminescence readings. Twenty-five microliters of CellTiter-Glo luminescence cell viability assay (Promega) were added to each well using the Multidrop Combi liquid handler. The plates were shaken on an orbital microplate shaker (VWR) at 360 rpm for 10 min, and then they were left for an additional 10 min before luminescence measurements on an EnVision 2104 Multilabel Reader (Perkin Elmer). The luminescence reading data were processed through Excel to determine growth inhibition percentages (17).

**(iii) Cysticidal assay.** Twenty-nine fractions displaying >90% inhibition were selected for screening against cysts. 5 × 10^3^ trophozoites were seeded in 99 μL of encystation medium into a 96-well black, clear-bottom microplate (Corning 3603) and incubated for 48 h to encyst the cells.

Afterward, 1 μL of each fraction or control was added to the cysts in duplicate. 1% (vol/vol) DMSO and 923.71 μM PHMB served as negative-controls and positive-controls, respectively. After 48 h of incubation at 28°C and 5% CO_2_, the medium was removed and washed four times with 100 μL PBS. Each well was then replaced with 100 μL of PYG growth medium and incubated for imaging.

After 3 days of incubation, each well of the plate was imaged on the ImageXpress Micro XLS. The images were converted from tiff to jpg images with a custom python script and run through the YOLOv3 algorithm to determine the counts of trophozoites on images taken 0 days and 3 days after switching to the PYG growth medium. The trophozoite counts from the positive and negative-control wells were used to generate a logistic regression, which was used to determine the cysticidal likelihood of each fraction. Duplicate wells displaying an average probability >0.9 of being cysticidal were considered hits. Additionally, the day 3 images were also manually verified to determine if the predictions were accurate. Because this was used as a filtering method to identify if a treatment was cysticidal or not, it was only performed once with technical replicates.

**(iv) EC_50_ determination and mammalian cytotoxicity assay.** The top 10 trophocidal compounds identified previously in the primary amebicidal screen were evaluated for their EC_50_ values. These fractions were serially diluted 2-fold (60 μg/mL to 468.75 ng/mL) using the BioSero ATS and 0.5 μL of each fraction was added to a Greiner Bio-One Cellstar white, flat bottom microplate. Afterward, 100 μL of PYG growth medium containing 5 × 10^3^ trophozoites was added via the Multidrop Combi liquid handler.

These plates were allowed to incubate for 48 h before CellTiter-Glo luminescence readings on the EnVision 2104 Multilabel Reader. Data from a minimum of three independent experiments (biological replicates) conducted in triplicate were analyzed on GraphPad Prism 6 to determine EC_50_ values.

For cytotoxicity assays, 0.5 μL of the selected fraction was transferred into a 96-well screen plate followed by the addition of 99.5 μL of HEK-293 or HT-29 cells (10,000 cells) to yield different concentrations of the fraction with a final DMSO concentration of 0.5%. The negative-control contained 0.5% DMSO and the positive-control contained 100 μM staurosporine (BioVision). The assay was performed in triplicate using the CellTiter-Glo Luminescent Cell Viability Assay (18).

### Determination of minimum cysticidal concentration of 12FP47A9.

Fraction 12FP47A9 was evaluated for a minimum cysticidal concentration. A plate of A. castellanii cysts was prepared following methods described in previous sections with minor alterations. To evaluate higher concentrations of 12FP47A9, 5 × 10^3^ trophozoites were encysted in 96, 98, and 99 μL of medium to achieve final concentrations ranging from 480 to 120 μg/mL. Additionally, 5 × 10^3^ trophozoites were also encysted in 99.5 μL of medium to achieve final concentrations ranging from 60 to 3.75 μg/mL. 4% (vol/vol) DMSO and 3,694.8 μM PHMB served as negative-controls and positive-controls, respectively. The experiment was repeated three times with biological and technical replicates on different days. Mature cysts were incubated with fraction 12FP47A9 for 48 h before the exchange with the PYG growth medium. Once in the PYG medium, the cysts were imaged by the ImageXpress Micro XLS, and images were manually evaluated for excystation at 7 days (17).
